# How Does Internet Use Affect the Farmers’ Trust in Local Government: Evidence from China

**DOI:** 10.3390/ijerph20043489

**Published:** 2023-02-16

**Authors:** Suhao Wei, Yangxiao Lu

**Affiliations:** 1 School of Public Administration, Jilin University, Changchun 130012, China; 2 College of Economics and Management, Jilin Agricultural University, Changchun 130118, China

**Keywords:** internet use, local government trust, livelihood problems, government performance evaluation

## Abstract

The rapid spread of the internet in rural China in the 21st century has fundamentally reshaped the operation of the Chinese rural political system in ways that are at least as profound as television half a century ago. This study used the data of 8754 farmers from 2018 China Family Panel Studies (CFPS) in China to examine and provide empirical evidence on how internet use affects farmers’ trust in local government via a chain-mediation model. The results indicate that internet use erodes farmers’ trust in local government. Internet use is more likely to cause young and highly educated farmers to lose trust in local government. Both views on people’s livelihood problems and government performance evaluations play mediating roles between internet use and farmers’ trust in local government. Further, we also found that the negative direct impact of internet use on farmers’ trust in local government is also serial mediated by views on people’s livelihood problems and government performance evaluations. The results expand the research on the factors influencing trust in government.

## 1. Introduction

A high level of political trust promotes economic and social development by influencing political participation, regulatory compliance, policy compliance, etc. [1,2,3]. Conversely, a low level of political trust seriously restricts the implementation of government policies, which is often an important reason for regime overthrow [4]. Therefore, in democracies and authoritarian regimes alike, maintaining a high level of public trust in government has always been an important goal of all incumbent administrations [5]. However, public trust in government and politics has been on a downward trend in most Western countries over the past few decades [6,7]. The studies state that the media, which are not controlled by the ruling party, are largely responsible for the decline in public trust in government [8]. Government trust is less threatened in countries where the media is controlled [9].

The level of Chinese people’s trust in government is among the highest in the world [10]. There are two possible reasons: first, the efforts of the Communist Party of China (CPC) in political and economic reforms have made people very satisfied with the government’s performance [11]. Under the leadership of the CPC, China has created a miracle of economic development. Unprecedented economic growth and income growth are the basis for the public to maintain a high level of trust in government [12]. Second, Confucian culture prevails in China, and Confucianism emphasizes absolute obedience to authority, which helps to improve Chinese people’s trust in government [13]. However, some studies claim that China’s political trust has shown a downward trend in recent years, and how to maintain a high level of political trust will be a huge challenge for the Chinese government [14,15].

The internet has made it easier than ever for citizens to access political information [16]. Since the beginning of the 21st century, China has achieved great success in rural informatization. Since 2005, 18 consecutive No. 1 documents have pointed out the need to promote rural informatization. By 2020, more than 98% of rural administrative municipalities in China were connected by fiber-optic and 4G networks, and the number of farmers using the internet had reached 309 million. The rapid spread of the internet in rural China, just as television did half a century ago, is fundamentally reshaping the operation of the political system. Unquestionably, the internet has brought massive amounts of information and opportunities for farmers to interact with readers, and has brought revolutionary changes to traditional information media [5]. However, internet use also poses tremendous challenges for censorship. With the great success of rural informatization, has internet use at least partially contributed to the decline of Chinese farmers’ trust in the government?

Does internet use have an impact on political science? Scholars have conducted extensive research. Although works in the literature propose plausible hypotheses and provide empirical evidence, they draw conflicting conclusions. On one hand, some works in the literature contend that the internet is more convenient to obtain information than traditional media, which improves the public′s level of political knowledge and promotes political participation [17]. Compared with traditional media, the internet also allows readers to interact, which helps spread political knowledge [18]. On the other hand, some studies assert that the internet may spread false information, including exaggerations of corruption, inequality, mistakes by government, etc., and induces internet users to make mistaken political decisions [19,20]. Especially, some social media relying on the internet have accelerated the spread of rumors, fake news and conspiracy theories [21]. Therefore, internet use does nothing to increase political knowledge and even damages political trust [22].

The debate over the positive and negative effects of internet use on political psychology continues to this day. However, neither of these theories is likely sufficient to explain the impact of internet use on Chinese farmers’ government trust. First, as a historical continuation, Chinese farmers continue to maintain a high level of trust in government [23]. Although, in the 21st century, the internet is rapidly spreading across rural China, there is still no answer to whether the use of the internet can shake farmers’ trust in government. Second, the Chinese government has invested heavily in managing the internet [24]. In 2011, the Chinese government established the State Internet Information Office (SIIO), and in 2018, the Cyberspace Administration of China directly under the Central Committee of the CPC was approved for establishment. Therefore, internet use may not lead to the spread of fake news, rumors and conspiracy theories. The level of public trust in the central government in China is higher than that in local government, showing a public trust level characteristic of “strong central and weak local”. Possible reasons are that (ⅰ) Confucian culture emphasizes obedience to the highest authority [14], (ⅱ) when implementing central policies, local governments sometimes deviate from the central government’s policy intentions [5], and (ⅲ) the central government is more representative of the public interest [25]. Farmers have more contact with local government in their production activities; therefore, we were more concerned about the relationship between internet use and farmers’ trust in local government.

To address this concern, we used CFPS data in 2018 to investigate the impact of internet use on farmers’ trust in local government. Moreover, this study examines the chain mediating effect of views on people’s livelihood problems and government performance evaluations. This paper also examines the following issues: (1) Does internet use affect farmers’ trust in local government? (2) Do the views on people’s livelihood problems and government performance evaluations mediate the relationship between internet use and farmers’ trust in local government? (3) Do views on people’s livelihood problems and government performance evaluations have a sequential mediation effect? Our research not only can expand the research on the factors influencing government trust, but also provide empirical evidence for assessing the impact of internet use on political psychology.

## 2. Theories and Hypotheses

### 2.1. Internet Use and Farmers’ Trust in Local Government

Trust is an important factor controlling individual behavior. Government trust reflects the public’s psychological judgment on the credibility of the government’s protection and promotion of public interests [26]. Therefore, the psychological state of trust and distrust depends on the gap between expectations and perceptions of the government. The psychological state of government trust reflects the degree to which the government’s governing concept, behavior and public interest expectations fit. The objects of government trust include specific government officials, specific government institutions, public policies formulated by the government, government systems, etc. [27].

The CPC’s success began in rural China. Since the Autumn Harvest Uprising in 1927, Chinese farmers have maintained a high level of political trust in the CPC [28]. In 1978, China began to broadcast CCTV News on television. Before the popularization of the internet, TV news was an important channel for Chinese farmers to access political information. TV news (e.g., CCTV News) helps to increase farmers’ trust in the government [29]. However, with the rapid development of rural informatization in China, the internet has gradually become an important source of information for farmers. Although the Chinese government has increased its investment in internet monitoring [24], considering the positive role of the internet in disseminating political information, internet use still undermines the role of traditional social media in maintaining a high level of political trust among farmers.

Traditional media (e.g., TV news) pay less attention to social injustices, corruption of local government officials and political scandals, which may prompt people to seek alternative sources of information. According to data released by the National Bureau of Statistics of China, the income of urban residents in 2021 was 2.5 times that of farmers. Compared with urban residents, Chinese farmers have lower incomes, higher labor intensity, and worse working conditions [30]. Although farmers have increased their household income through off-farm employment, most of them are engaged in manual work such as construction, transportation, cleaning, etc. in cities and are more vulnerable to urban exclusion and unfair practices.

The internet makes it easier for farmers to obtain information about their own situation and social injustice. For example, internet social platforms such as WeChat, Douyin and Microblog pay more attention to the status of farmers and vulnerable groups. Rumors, conspiracies, and false information spread through online social media three times faster than normal information [21]. Surprisingly, Chinese citizens have higher levels of trust in online information. Moreover, efforts to correct or debunk misinformation on internet sites often prove to be futile, even backfiring and reinforcing misunderstandings [31]. Chinese internet users are more concerned about unfair incidents that can trigger the rapid spread of information on cases of farmer inequality over the internet. The information obtained by farmers through the internet is inconsistent with the information promoted by the government media and may trigger farmer boycotts and distrust of the official media, and even discredit official information sources. Therefore, internet use likely damages farmers’ trust in local government. Thus, we propose the following hypothesis:

**H1:**  *Internet use is negatively associated with local government trust in rural China.*

### 2.2. The Mechanism of Internet Use Affecting Farmers’ Trust in Local Government

The internet reduces the cost of farmers’ access to information; this has led to changes in farmers’ views on people’s livelihood problems. Traditional official media pay less attention to social inequality and the disadvantaged position of farmers [32], while internet social platforms pay more attention to the plight of farmers. As mentioned earlier, internet social platforms such as WeChat, Douyin and Microblog pay more attention to the status of farmers and vulnerable groups. Therefore, compared with the traditional official media, internet use makes farmers more aware of their situation, especially the people’s livelihood problems. The internet allows farmers to obtain more information about education, medical care, housing, income gaps, corruption and other matters. Through information acquisition, farmers may be more inclined to think that the gap between their education levels and those of urban residents is larger, their medical conditions are worse, and their income gap with urban residents is larger. Farmers who use the internet tend to think that people’s livelihood problems in China are more serious. Individual ideas are important factors that affect individual political confidence, attitude and behavior. If farmers think that China’s livelihood problems are more serious, they are more likely to lose confidence in the local government, thereby reducing the level of trust. Thus, we propose the following hypothesis:

**H2:**  *Views on people’s livelihood problems mediate the relationship between internet use and farmers’ trust in local government.*

Internet use not only allows farmers to understand the operation of local government in an unprecedented way by sharing rich information, but also enables farmers to learn more about the actual performance of local government through interactive communication [33]. Greater knowledge of local government may raise farmers’ expectations of local government performance. To a large extent, the rising expectations of citizens explain why political trust and support in industrialized countries continue to be undermined, while the failure of governments is less important [27]. Meanwhile, long-term high economic growth reduces the evaluation of the actual performance of the government [34]. Therefore, even if the performance of local government does not deteriorate, the high expectations brought by the internet will still lead to a decline in farmers’ evaluations of local government performance. Therefore, internet use increases farmers’ expectations for transparency and participation in government decision-making. Lower government performance evaluations will reduce farmers’ trust in local government. Thus, we propose the following hypothesis:

**H3:**  *Evaluations of local government performance mediate the relationship between internet use and farmers’ trust in local government.*

It is an important task for local governments to balance the equality of education, medical care, housing and other resources between farmers and urban residents. Farmers’ attitudes regarding people’s livelihood problems, including education, health care, housing, work and income gaps directly affect their evaluations of local government performance. Therefore, we expected that views on people’s livelihood problems and evaluations of local government performance are potentially important as sequential mediators that can explain the relationship between internet use and farmers’ trust in local government. Specifically, through information acquired over the internet, farmers may think that the livelihood problems related to themselves are more serious, expect the local government to solve their livelihood problems, and accordingly lower their evaluations of local government performance. Thus, we propose the following hypothesis:

**H4:**  *The relationship between internet use and farmers’ trust in local government is sequentially mediated by the views on people’s livelihood problems and evaluations of local government performance.*

## 3. Data and Methods

### 3.1. Data Source

In this paper, the data used for statistical analysis came from the 2018 CFPS, which was conducted by the Institute of Social Science Survey at Peking University. Since it is designed following the Panel Study of Income Dynamics (PSID), the CFPS is often called the Chinese PSID. The 2018 CFPS covered 25 provinces and thus could be regarded as a representative sample of China. The CFPS questionnaire covers household characteristics, demographics, politics, income, education, and the health of respondents—data that are applicable to research on the relationship between internet use and farmers’ trust in local government in China.

The CFPS adopted a multistage stratification with a probability proportional to size (PPS) sampling strategy and divided the whole population into six sampling frames aimed at generating national representative and provincial representative samples. The sampling procedure involved three stages. First, 144 counties and 32 county-level streets (townships) were randomly selected according to per capita GDP. Second, specific communities or villages were select at random from sample counties. Third, in the sampled communities and villages, the end sampling frame was constructed using the map address method, and the sample farmers were randomly selected. The 2018 CFPS covered 33,326 adult respondents in 14,241 families from 25 provinces.

The strength of the 2018 CFPS was its focus on internet access and political trust, which provided a rare opportunity to identify details on internet use, attitudes toward livelihood problems, government trust, and other personal characteristics. Since this article was focused on farmers’ trust in local government, we only used adult respondents over the age of 16 who lived in rural communities. It should be noted that we eliminated the samples missing important data and finally included 8754 farmers in our sample.

### 3.2. Variables

The dependent variable in this study is farmers’ trust in local government. Public trust in government officials is usually used as a token variable of government trust [5]. After China abolished the agricultural tax in 2006, the power of township government was greatly weakened. Although, the township government is the lowest level of government in China, the dispatched offices of the county government occupy some of the functions of the township government. Therefore, we use farmers’ trust in county-level government officials to represent farmers’ trust in local government.

Internet use was the main independent variable. We constructed a measure for internet use as follows: (ⅰ) if farmers used the internet via mobile phone or computer (at least one kind), we defined those farmers as using the internet and assigned them a value of 1; (ⅱ) otherwise, farmers who did not use the internet were assigned a value of 0. The data show that 66.95% of farmers reported the experiences of internet use via mobile phone or computer.

Views on people’s livelihood problems and evaluations of local government performance were the mediating variables. We used farmers’ views regarding livelihood problems to express their views on people’s livelihood problems, including the wealth gap, employment, environmental protection, education, medical care, social security, housing and government corruption. Farmers’ concerns about these eight livelihood problems were all treated as multi-valued ordinal variables ranging from 0 to 10, where 0 meant very serious and 10 meant not serious. We added up the eight livelihood issues to obtain the value for farmers’ views on people’s livelihood problems, and the smaller the value was, the more serious that farmers thought people’s livelihood problems were. We used farmers’ views on the actual performance of county-level government to express their evaluations of local government.

In the analysis of the impact of internet use on farmers’ trust in local government, other variables controlled in this article included individual, family and location characteristics. First, a person’s individual characteristics shape that person’s personality, values and attitudes, which can explain some of the differences in a person’s trust in government [17,35]. Therefore, we controlled for the variables of individual characteristics (e.g., gender, age, education, health, nationality, CPC member, non-agricultural employment (NFC), religion, trust in strangers). Second, family characteristics of respondents, especially income level, can affect respondents’ government trust [16]. Therefore, we controlled for the variables of family characteristics (e.g., per capita income, subsidy income, family size). Factors that cannot be observed in a region may have an impact on farmers’ trust in local government; thus, we needed to control the regional dummy variables. We divided all provinces into four regions: eastern, central, western, and northeastern, and constructed three regional dummy variables. Table 1 describes the model variables and summary statistics.

### 3.3. Empirical Strategy

The aim of the direct effect model was to assess the impact of internet use on farmers’ trust in local government. We adopted the following multiple linear regression model to build Model (1).

(1) $${\mathrm{Trust}}_{i}=\alpha_{0}+\alpha_{1}{\mathrm{Internetuse}}_{i}+\overset{n}{\underset{i=1}{\sum }}\beta_{j}X_{\mathrm{ij}}+\varepsilon_{i}$$

where ${\mathrm{Trust}}_{i}$ denotes farmers’ trust in local government. ${\mathrm{Internetuse}}_{i}\;$ represents internet use. $X_{\mathrm{ij}}$ are the control variables that include individual, family and location characteristics. $\alpha_{0}$ is a constant term. $\alpha_{1}$ and $\beta_{j}$ are the regression coefficients to be estimated. $\varepsilon_{i}$ is the random error, which is assumed to be independent and normally distributed.

However, in order to verify the robustness of the direct effect, we needed to consider the sample self selection error and endogenous error of Model (1). First, decisions to use the internet may not be random but intentional, which can lead to selection bias and inconsistent estimates. For example, farmers’ decisions regarding using the internet may be affected by others in the same village; hence, these farmers will have a higher probability of using the internet, which may result in self-selection bias. We used propensity score matching (PSM) to compensate for the impact of self-selection bias on estimation results.

The basic idea of PSM is to construct a counterfactual framework to eliminate self-selection bias. We divided farmers into two groups based on their decision to use the internet: farmers who used the internet were the treatment group, and farmers who did not use the internet were the control group. First, we used the probit model to predict the probability of farmers using the internet and estimated the propensity score value, second, performed matching based on propensity scores, and third, compared the average difference in farmers’ trust in local government between the treatment group and the control group and estimated the average treatment effect on the treatment group (ATT), as follows:

(2) $$\mathrm{ATT}=E\left\{E\left[\left(Y_{1i}|D_{i}=1,P\left(X_{i}\right)\right)\right]-E\left[\left(Y_{0i}|D_{i}=0,P\left(X_{i}\right)\right)\right]|D_{i}=1\right\}$$

where $D_{i}$ is a binary dummy variable reflecting whether a farmer can be assigned to the control group. If $D_{i}=1$, the farmer is assigned to the treatment group. If $D_{i}=0$, the farmer is assigned to the control group. $P\left(X_{i}\right)$ is the propensity score. $Y_{1i}$ represents the estimation result for the treatment group, and $Y_{0i}$ represents the estimation result for the control group.

Second, omitting important variables and reverse causality could confound our parameter estimations and, hence, the statistical inference. To some extent, the reverse causality of internet use on farmers’ trust in local government exists. Farmers with low-level trust in local government are generally skeptical of traditional media (e.g., TV news) and tend to use unofficial media for information, which means that they are more likely to use the internet. Although we controlled for variables such as a person’s individual, family and location characteristics, which may affect farmers’ trust in local government, we may have still missed some variables; for example, we could not accurately measure farmers’ values and outlooks on life.

We used the instrumental variable (IV) method to solve the above endogenous problem. The average monthly expenditure on communication, post and telecommunication (CPT) by farmers in the past 12 months was utilized to serve as the IV. The average monthly expenditure of farmers was CNY 0.232 thousand. For the exogenous and exclusion restrictions of the IV, first, mobile communication and computer network were important components of CPT, and farmers used the internet via mobile phones and computers. Therefore, the higher the expenditure on CPT, the higher the probability that farmers used the internet. Second, expenditure on CPT did not directly affect farmers’ trust in local government, but only affected their internet use. The endogenous variable was a binary indicator; the two-stage least squares method (2SLS) could not be used to estimate the model parameters. We used the conditional mixed process (CMP) instead. CMP allows continuous, binary and ordered endogenous variables.

Third, we needed to test the mediation effect of views on people’s livelihood problems. We build mediating effect Models (3) and (4) based on direct effects.

(3) $${\mathrm{Livea}}_{i}=\gamma_{0}+\gamma_{1}{\mathrm{Internetuse}}_{i}+\overset{n}{\underset{i=1}{\sum }}\delta_{j}X_{\mathrm{ij}}+\varepsilon_{i}$$

(4) $${\mathrm{Trust}}_{i}=\rho_{0}+\rho_{1}{\mathrm{Internetuse}}_{i}+\rho_{2}{\mathrm{Livea}}_{i}+\overset{n}{\underset{i=1}{\sum }}\theta_{j}X_{\mathrm{ij}}+\varepsilon_{i}$$

where ${\mathrm{Livea}}_{i}$ represents farmer’s views on people’s livelihood problems. $\gamma_{0}$ and $\rho_{0}$ are constant terms. γ1, $\delta_{j}$, $\rho_{1}$, $\rho_{2}$, $\theta_{j}$ are estimated parameters. The other variables are the same as those in Model (1).

Fourth, we needed to test the mediation effect of the evaluation of local government performance. We build mediating effect Models (5) and (6) based on direct effects.

(5) $${\mathrm{Evalua}}_{i}=\omega_{0}+\omega_{1}{\mathrm{Internetuse}}_{i}+\overset{n}{\underset{i=1}{\sum }}\varphi_{j}X_{\mathrm{ij}}+\varepsilon_{i}$$

(6) $${\mathrm{Trust}}_{i}=\epsilon_{0}+\epsilon_{1}{\mathrm{Internetuse}}_{i}+\epsilon_{2}{\mathrm{Evalua}}_{i}+\overset{n}{\underset{i=1}{\sum }}\mu_{j}X_{\mathrm{ij}}+\varepsilon_{i}$$

where ${\mathrm{Evalua}}_{i}$ represents farmers’ evaluation of local government performance. $\omega_{0}$ and $\epsilon_{0}$ are constant terms. $\omega_{1}$, $\varphi_{j}$, $\epsilon_{1}$, $\epsilon_{2}$, $\mu_{j}$ are estimated parameters. The other variables are the same as those in Model (1).

Fifth, we needed to test the chain mediating effect of the views on people’s livelihood problems and the evaluation of local government performance. We build the chain mediating effect Models (7) and (8) based on direct effects.

(7) $${\mathrm{Evalua}}_{i}=\tau_{0}+\tau_{1}{\mathrm{Internetuse}}_{i}+\tau_{2}{\mathrm{Livea}}_{i}+\overset{n}{\underset{i=1}{\sum }}\sigma_{j}X_{\mathrm{ij}}+\varepsilon_{i}$$

(8) $${\mathrm{Trust}}_{i}=\vartheta_{0}+\vartheta_{1}{\mathrm{Internetuse}}_{i}+\vartheta_{2}{\mathrm{Evalua}}_{i}+\vartheta_{3}{\mathrm{Livea}}_{i}+\overset{n}{\underset{i=1}{\sum }}v_{j}X_{\mathrm{ij}}+\varepsilon_{i}$$

The causal steps method is a common approach for testing mediation effects. However, when the mediating effect is weak, the causal steps method cannot detect a significant mediating effect [36]. The Sobel test is considered as an important alternative to the causal steps method by directly testing mediation effects. However, the accuracy of the Sobel test is also questioned. The bootstrap method is a more accurate test approach and has gradually replaced the causal steps method and Sobel test. Therefore, in order to ensure the robustness of the mediation effects test results, we used the causal steps method, Sobel test, and bootstrap method to test mediation effects at the same time.

## 4. Results

### 4.1. Impacts of Internet Use on Farmers’ Trust in Local Government

Table 2 reports the parameter estimates for Model (1). The results in columns (1) and (2) were estimated using the Oprobit model and OLS, respectively. Columns (3) and (4) present results of the first-stage and second-stage regression for CMP, respectively. The Atanhrho_12 test in column (4) indicated the presence of the endogenous problem. In the first-stage results, the coefficients on the IV method passed the significance test at the 1% level, which indicated that the IV method was available.

The results in columns (1), (2) and (4) all showed that internet use has a negative impact on farmers’ trust in local government at the 1% significance level, indicating that farmers who use the internet present less trust in local government than farmers with no internet use. The coefficient of internet use in column (4) was larger than that in column (2), indicating that the negative impact of internet use on farmers’ trust in local government was greater when the endogenous problem was considered. The results in Table 2 support our Hypothesis 1. Before the popularization of the internet, information channels in rural China were limited to traditional media. However, the results in Table 2 indicate that, with the rapid advancement of informatization in rural China, the internet has gradually replaced the traditional media and has become a new channel for farmers to obtain information, undoubtedly posing unprecedented challenges to farmers’ trust in local government.

Among the other control variables, women trusted local government more than men. This may be related to gender inequality in internet use [37]. Older age was associated with higher levels of trust in local government, which supported the viewpoint that young people are skeptical of politics. Farmers with better self-reported health condition trusted the local government more. Farmers with poor health were often in poverty and had higher expectations of local government performance. Therefore, they had lower confidence in local government. Farmers with non-farm employment experience obtained more information on income gaps and inequality, which damaged farmers’ trust in local government. Farmers with religious belief have a higher level of trust in local government. A possible reason was that religious belief helped to reduce immoral, illegal behavior and doubts about political power [38]. Farmers who were more likely to trust strangers were also more likely to trust local government, which was consistent with common sense. Government subsidies helped increase farmers’ trust in local government. In addition, we found that currently divorced or married farmers tended to trust government less. Possible reasons were s that divorced farmers in rural China are more likely to suffer discrimination and economic difficulties, and married farmers are under greater financial pressure. The other control variables had insignificant impacts on farmers’ trust in local government.

### 4.2. Heterogeneity Analysis: Who Is More Likely to Lose Trust in Local Government?

Table 3 presents the results of the heterogeneity analysis, which were estimated using the Oprobit model. The results in columns (1) and (2) were estimates for samples of farmers born before and after 1978, respectively, which helped to test whether a “digital divide” in age levels exists. The results in columns (3) and (4) were estimates for samples of farmers who had a complete or incomplete 9-year compulsory education, respectively, which helped to test whether a “digital divide” in education levels exists.

The results in columns (1) and (2) indicated that internet use by farmers born after 1978 had a greater negative impact on local government trust than farmers born before 1978. The impact of internet use on trust in local government was unevenly distributed among farmers, supporting the argument of “digital divide”. Farmers born before 1978 had experienced the movement of people’s communes, the Great Leap Forward, and the Cultural Revolution. These political events helped them maintain a high level of political trust in local government. Moreover, early political events were an important source of accumulated political knowledge in the early lives of farmers that affected them throughout their lives. However, farmers born after 1978 did not experience these political events. In addition, young people tended to learn and use the internet more easily than older people.

The results in columns (3) and (4) indicated that internet use had a greater negative impact on trust in local government among farmers who had completed the 9-year compulsory education compared to those who had not completed the 9-year compulsory education, thus also supporting the argument of a “digital divide”. Farmers with higher levels of education could learn the internet faster and obtain more information. They were also more concerned about inequality, income disparity, corruption, etc. Therefore, they were more likely to lose trust in local government under the influence of the internet. It should be noted that control variables did not change significantly from Table 2, so we no longer interpreted the results for the control variables.

### 4.3. Robustness Check

**Figure 1:**
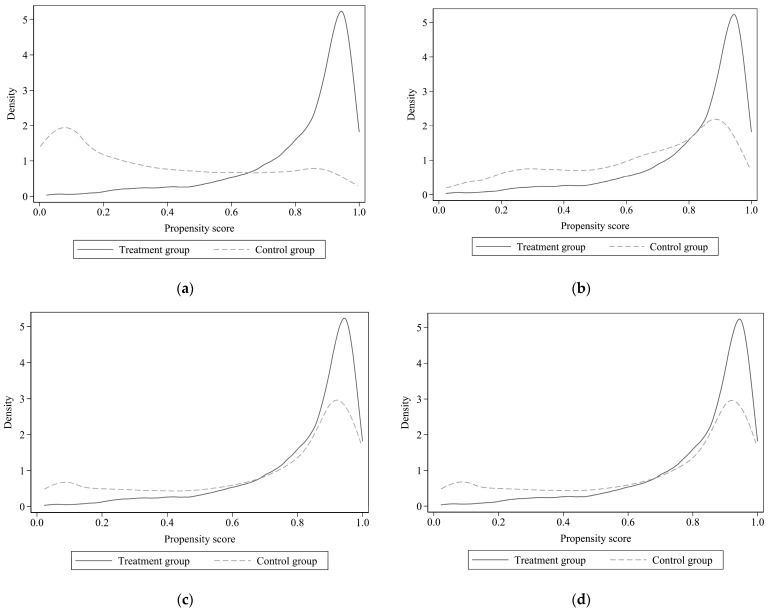
Probability distributions of propensity score between treatment and control groups before and after matching for Model (1). (**a**) Before matching, (**b**) nearest neighbor (1 to 4), (**c**) radius (caliper), (**d**) kernel.

Here, we ran an array of robustness checks. First, self-selection bias was the main source of estimation bias in this study. We used PSM to re-estimate Model (1). Five matching strategies—nearest neighbor (1 to 1), nearest neighbor (1 to 4), radius (caliper), kernel and local linear regression—were used to estimate the ATT. Figure 1 shows the probability distributions of the propensity score between treatment and control groups before and after matching. According to Figure 1, the difference between the treatment group and the control group was large before matching, but the difference was reduced after matching. Therefore, the matching effect was good. Table 4 reports the results of PSM estimation. Farmers who used the internet were likely to have lower trust in local government than farmers with no internet access, as the ATT was significantly negative. In general, we can conclude that self-selection bias or omitted variables did not confound our estimates.

Second, to distinguish the amount of time farmers spent on the internet, we created an alternate measure and replaced the dummy variable with the amount of time farmers used the internet in their spare time per week. Farmers used the internet for an average of 9.73 h a week in their spare time. We estimated the impact of the internet usage time on farmers’ trust in local government. Column (1) in Table 5 reports the estimated results. The results show that increasing internet usage time was likely to decrease farmers’ trust in local government, thus still supporting our conclusion.

Third, although our sample covered almost the whole of China, the level of agricultural development varied widely across provinces. The CFPS divides Chinese provinces into large and small provinces, and the sampling ratio of large provinces is higher than that of small provinces. Henan is the largest wheat producing province in China. Among the samples used in this article, the number of samples from Henan was 1550, representing the province with the largest sample size. As a robustness test, this article re-estimated the impact of internet use on farmers’ trust in local government using the samples from Henan. Column (2) in Table 5 reports the estimated results. The results show that internet use still negatively affected farmers’ trust in local government.

### 4.4. Mechanisms

Table 6 presents the parameter estimates for Models (3)–(6). Columns (1) and (2) present the mediation effect test results for the views on people’s livelihood problems. First, the coefficient of internet use was negative in column (1) for the causal steps method and passed the significance test at the 1% level, indicating that farmers who use the internet believe that people’s livelihood problems in China are more serious. The results in column (2) show that the coefficients of internet use and the views on people’s livelihood problems passed the significance test at the level of 1% after incorporating them into the model. According to the results of the causal steps method, we determined that the mediation effect of the views on people’s livelihood problems was established, and the mediation effect was (−2.813)×0.011=−0.031. Second, the mediation effect of the Sobel test passed the significance test at the 1% level, and the mediation effect was −0.031. Third, the mediation effect of the bootstrap model passed the significance test at the 1% level, the mediation effect was −0.031, and the 95% confidence interval did not contain 0. Therefore, the results for the causal steps method, Sobel test and bootstrap model were consistent, supporting Hypothesis 2.

Columns (3) and (4) present the mediation effect test results for local government performance evaluation. The coefficient of internet use was negative in column (3) for the causal steps method and passed the significance test at the 1% level, indicating that farmers who use the internet have lower evaluations of local government performance. The results in column (4) show that the coefficients of internet use and evaluation of local government performance passed the significance test at the level of 1% after incorporating them into the model. According to the results for the causal steps method, we determined that the mediation effect of local government performance evaluation was established, and the mediation effect was (−0.037)×0.978=−0.036. The mediation effects in both the Sobel test and bootstrap model passed the significance test at the level of 5%. The results for the causal steps method, Sobel test and bootstrap model were consistent, supporting Hypothesis 3.

Table 7 presents the results for the chain mediating effect of the views on people’s livelihood problems and the evaluation of local government performance in Models (7) and (8). The coefficient of the views on people’s livelihood problems passed the significance test at the 1% level in column (1). The coefficient of the evaluation of local government performance passed the significance test at the 1% level in column (2). The chain mediating effect of the views on people’s livelihood problems and the evaluation of local government performance was (−0.290)×0.005×0.968=−0.001. Therefore, internet use had a negative impact on farmers’ trust in local governments through the chain mediating effect of “the views on people’s livelihood problems→the evaluation of local government performance”, supporting Hypothesis 4.

## 5. Discussion

The results of our study have important implications for governance in rural China. As more and more farmers use the internet, farmers’ political trust is intentionally or unintentionally affected by the internet. In view of the negative impact of internet use on farmers’ trust in local government, curbing the loss of government trust in rural China is a pressing issue in the current social economy. The findings of this paper provide new ideas to address this issue. First, the Chinese government should moderately strengthen its intervention in internet information and ensure that the information about government performance on the internet is consistent with the information published by the traditional media. Second, the Chinese government should pay more attention to the provision of public services, solve the livelihood problems that farmers care about, and commit to improving government performance. Particularly, local government should address urban–rural inequality, increase the supply of rural public services, and intensify anti-corruption efforts to restore farmers’ confidence. Third, the government should devote more efforts to curbing the loss of government trust among farmers born after 1978 or with higher levels of education.

This study makes the following contributions. First, previous research on the political consequences of internet use has focused on the access to political knowledge and information and the impact on political participation by different groups, but has provided little evidence on the association between internet use and farmers’ trust in local government. This study addresses this gap and documents systematic evidence on whether and how internet use can influence farmers’ trust in local government. Second, although a large body of literature has explored the impact of traditional media on political trust, less attention has been paid to the impact of internet use on government trust. Particularly, there is a lack of research on the impact of internet use by Chinese farmers, a traditionally vulnerable group. This paper contributes to existing studies by exploring the impact of internet use on farmers’ trust in local government and its mechanisms in China. Third, after separating different groups, this paper provides systematic evidence of the asymmetric impact of internet use on farmers’ trust in local government.

Our study, of course, has its limitations. This article only examined the impact of the dummy variables of internet access and internet usage time on farmers’ trust in local government. However, the pattern of internet use by farmers is constantly changing. More and more Chinese farmers use social media platforms such as WeChat and Douyin to obtain information. For example, the shooting and uploading of small videos has become a new mode for farmers’ interaction and information dissemination. Due to data limitations, this article could not test the impact of social media platforms on government trust. In addition, the government’s supervision of internet information is also changing quietly. In closing, we call for more detailed studies given the vast differences in social media platforms. In order to deeply explore the impact of internet use on farmers’ trust in local government, more experimental research is needed on social media such as WeChat and Douyin when data are available.

## 6. Conclusions

With the rapid advancement of Chinese rural informatization projects, the internet has increasingly become an important source of information for Chinese farmers in addition to traditional media. Therefore, the importance of understanding the political consequences of internet use by Chinese farmers cannot be overemphasized. The ease of access to information and the difficulty of implementing supervision are important features that differentiate the internet from traditional media. As a continuation of history, Chinese farmers have long maintained a higher level of trust in government than urban residents. However, internet use is challenging the high level of government trust in rural China. Although many works in the literature have explored the impact of traditional media on political trust, few have paid attention to the impact of internet use on government trust in rural China. This paper contributes to the existing studies by exploring the impact of internet use on local government trust in rural China.

This article theoretically analyzed the impact of internet use on farmers’ local government trust and its mechanisms and then empirically tested the impact of internet use using data by from the 2018 CFPS on 8754 farmers in China. The results indicate that internet use undermines farmers’ trust in local government. The results of the heterogeneity discussion show that internet use is more likely to cause young farmers who have not experienced rural political events and farmers with higher education to lose trust in local government, which aligns with the “digital divide” argument. This study also found that internet use can erode farmers’ trust in local government by causing adverse changes in farmers’ views on people’s livelihood problems and reducing farmers’ evaluations of local government performance. Moreover, internet use has a negative impact on farmers’ trust in local government through the chain mediating effect of “the views on people’s livelihood problems→the evaluation of local government performance”.

## Figures and Tables

**Table 1:** Variable definitions and summary statistics of sample.

Variable Name	Definition	Mean	SD
Trust in local government	Farmers’ trust in county-level government officials, value 0 to 10; 0 = completely distrust, 10 = completely trust	5.073	2.618
Internet use	1 if farmers use the internet via mobile phone or computer (at least one kind), and 0 otherwise	0.670	0.470
Views on people’s livelihood problems	Values 0 to 80, 0 = very serious, 80 = not serious	26.749	15.148
Evaluation of local government performance	Farmers’ evaluations of the actual performance of county-level governments; 1 = no achievement, 2 = little achievement, 3 = certain achievement, 4 = great achievement	2.461	0.850
Gender	1 if the farmer is male, and 0 otherwise	0.493	0.500
Age	Age of the farmer (years)	49.968	21.321
Education ^a^	1 = uneducated, 2 = elementary school, 3 = junior high school, 4 = high school/secondary school/technical school/vocational high school, 5 = junior college, 6 = college and above	2.757	1.277
Health	Farmer’s self-identified health: 1 = unhealthy, 2 = average, 3 = quite healthy, 4 = very healthy, 5 = exceptionally healthy	3.262	1.178
Marriage ^b^	1 = unmarried, 2 = married, 3 = divorced, 4 = widowed	1.823	0.573
Nationality	1 if the farmer belongs to minority nationality, and 0 otherwise	0.014	0.118
CPC member	1 if the farmer is a member of the CPC, and 0 otherwise	0.013	0.112
NFC	1 if the farmer has non-agricultural employment experience, and 0 otherwise	0.678	0.467
Religion ^c^	1 if the farmer has religious beliefs, and 0 otherwise	0.390	0.488
Trust in strangers	Farmers’ trust in strangers, value 0–10; 0 = very distrustful, 10 = very trusting	2.353	2.190
Per capita income	Per capita income of farm household in the past 12 months (CNY 10,000)	1.572	2.133
Per capita subsidy income	Government subsidies per person in farm household in the past 12 months (CNY 10,000)	0.040	0.218
Farm population	Number of farm household	4.592	2.187
Region dummy variables	Eastern, central, western and northeast of China	—	—

Note(s): ^a^ Illiterate and semi-literate farmers usually have no formal education, so we included them into the uneducated group. ^b^ The sample size for cohabitation (unmarried) was only 27, so we removed the marital status of cohabitation (unmarried). ^c^ If a farmer had one or more beliefs including Buddha, Bodhisattva, immortals, Allah, God, or Jesus, we defined that farmer as having religious beliefs.

**Table 2:** Impacts of internet use on farmers’ trust in local government.

Variable Name	(1) OLS	(2) Oprobit	(3) CMP Fist-Stage	(4) CMP Second-Stage
Internet use	−0.290 *** (0.078)	−0.119 *** (0.031)		−0.270 *** (0.098)
IV for internet use			0.142 * (0.079)	
Gender	−0.267 *** (0.055)	−0.105 *** (0.023)	0.089 ** (0.036)	−0.100 *** (0.023)
Age	0.021 *** (0.003)	0.008 *** (0.001)	−0.055 *** (0.002)	0.006 *** (0.002)
Education	−0.018 (0.028)	−0.009 (0.012)	0.374 *** (0.019)	0.005 (0.015)
Health	0.172 *** (0.026)	0.070 *** (0.010)	−0.030 ** (0.015)	0.069 *** (0.010)
Nationality	0.180 (0.235)	0.071 (0.094)	−0.220 (0.137)	0.062 (0.094)
CPC member	−0.028 (0.244)	−0.002 (0.098)	0.048 (0.176)	−0.004 (0.098)
NFC	−0.188 *** (0.071)	−0.076 *** (0.027)	0.189 *** (0.039)	−0.064 ** (0.028)
Religion	0.092 (0.057)	0.040 * (0.023)	−0.022 (0.036)	0.038 * (0.023)
Trust in strangers	0.286 *** (0.013)	0.118 *** (0.005)	−0.001 (0.008)	0.118 *** (0.005)
Per capita income	−0.045 *** (0.011)	−0.018 (0.026)	0.066 (0.113)	−0.017 (0.026)
Per capita subsidy income	0.200 (0.143)	0.081 (0.050)	0.055 (0.089)	0.083 * (0.050)
Family population	0.010 (0.014)	0.004 (0.006)	−0.017 * (0.009)	0.004 (0.006)
Marriage (taking “unmarried” as reference)				
Married	−0.760 *** (0.077)	−0.305 *** (0.033)	0.422 *** (0.056)	−0.285 *** (0.035)
Divorced	−0.513 ** (0.212)	−0.201 ** (0.082)	0.561 *** (0.125)	−0.175 ** (0.084)
Widowed	−0.170 (0.226)	−0.051 (0.087)	−0.043 (0.179)	−0.033 (0.087)
Region dummy		yes	yes	yes
Constant	4.133 *** (0.222)		1.221 *** (0.135)	
Observations	8754	8754	8754	8754
Atanhrho_12				0.089 * (0.048)
Pseudo R2	0.085	0.021		

Note: Robust standard errors are presented in parentheses; *, **, and *** indicate significance levels at 10%, 5%, and 1%, respectively.

**Table 3:** Results of heterogeneity analysis.

Variable Name	Birth Date		9-Year Compulsory Education	
	(1) Before 1978	(2) After 1978	(3) Complete	(4) Incomplete
Internet use	−0.065 *** (0.019)	−0.246 *** (0.051)	−0.197 *** (0.064)	−0.084 ** (0.035)
Gender	−0.107 *** (0.027)	−0.063 (0.042)	−0.100 * (0.052)	−0.098 *** (0.026)
Age	0.006 ** (0.003)	0.011 *** (0.002)	0.007 *** (0.002)	0.008 *** (0.002)
Education	−0.018 (0.013)	0.027 (0.026)	-	−0.010 (0.014)
Health	0.086 *** (0.013)	0.041 *** (0.016)	0.054 *** (0.019)	0.076 *** (0.012)
Nationality	−0.050 (0.111)	0.419 ** (0.178)	0.088 (0.144)	0.008 (0.126)
CPC member	−0.015 (0.110)	0.004 (0.222)	−0.029 (0.455)	0.004 (0.101)
NFC	−0.080 ** (0.037)	−0.116 *** (0.043)	−0.112 ** (0.052)	−0.062 * (0.033)
Religion	0.021 (0.028)	0.094 ** (0.041)	0.141 *** (0.049)	0.004 (0.026)
Trust in strangers	0.133 *** (0.007)	0.097 *** (0.009)	0.097 *** (0.011)	0.125 *** (0.006)
Per capita income	−0.022 (0.026)	−0.004 (0.013)	−0.008 (0.020)	−0.023 (0.026)
Per capita subsidy income	0.046 (0.055)	0.272 ** (0.120)	0.080 (0.065)	0.096 *** (0.030)
Family population	0.001 (0.007)	0.013 (0.011)	0.036 *** (0.012)	−0.007 (0.006)
Marriage (taking “unmarried” as reference)				
Married	−0.180 *** (0.042)	−0.008 (0.128)	−0.240 ** (0.116)	−0.287 *** (0.036)
Divorced	−0.081 *** (0.025)	0.151 (0.179)	−0.112 (0.204)	−0.192 ** (0.092)
Widowed	0.369 (0.272)	0.141 (0.151)	−0.004 (0.148)	−0.068 (0.156)
Region dummy	yes	yes	yes	yes
Observations	6029	2725	2398	6356
Pseudo R2/R2	0.023	0.018	0.020	0.020

Note: Robust standard errors are presented in parentheses; *, **, and *** indicate significance levels at 10%, 5%, and 1%, respectively.

**Table 4:** Results of PSM estimation.

Matching Method	Treatment Group	Control Group	ATT
Nearest neighbor (1 to 1)	4.917	5.054	−0.137 *** (0.051)
Nearest neighbor (1 to 4)	4.917	5.101	−0.184 *** (0.035)
Radius (caliper)	4.917	5.096	−0.179 *** (0.030)
Kernel	4.917	5.036	−0.119 *** (0.016)
Local linear regression	4.917	5.094	−0.177 *** (0.051)

Note: Robust standard errors are presented in parentheses; *, **, and *** indicate significance levels at 10%, 5%, and 1%, respectively. The nearest neighbor matching was playback matching. The caliper was set to 0.01 in the radius (caliper) matching. The kernel function of kernel matching was a quadratic kernel function, and the bandwidth was set to 0.06. The local linear regression matching kernel function adopted three-three kernels, and the bandwidth was set to 0.8.

**Table 5:** Results for changing variables and samples.

Variable Name	(1) Oprobit	(2) Oprobit
Internet usage time	−0.002 ** (0.001)	
Internet use		−0.152 ** (0.066)
Gender	−0.110 *** (0.023)	−0.114 ** (0.055)
Age	0.010 *** (0.001)	0.003 (0.003)
Education	−0.016 (0.011)	0.009 (0.028)
Health	0.071 *** (0.010)	0.049 ** (0.024)
Nationality	0.074 (0.094)	−0.582 (0.395)
CPC member	−0.000 (0.098)	0.060 (0.225)
NFC	−0.081 *** (0.027)	−0.053 *** (0.015)
Religion	0.041 * (0.023)	−0.023 (0.056)
Trust in strangers	0.118 *** (0.005)	0.135 *** (0.013)
Per capita income	−0.018 (0.016)	−0.019 (0.014)
Per capita subsidy income	0.079 (0.050)	0.076 (0.118)
Family population	0.005 (0.006)	0.004 (0.015)
Marriage (taking “unmarried” as reference)		
Married	−0.322 *** (0.032)	−0.218 *** (0.079)
Divorced	−0.221 *** (0.082)	−0.290 (0.206)
Widowed	−0.064 (0.086)	0.391 (0.315)
Region dummy	yes	yes
Observations	8754	1550
Pseudo R2/R2	0.020	0.024

Note: Robust standard errors are presented in parentheses; *, **, and *** indicate significance levels at 10%, 5%, and 1%, respectively.

**Table 6:** The mediation effect of views on people’s livelihood problems.

Variable Name	(1) Views on People’s Livelihood Problems	(2) Farmers’ Trust in Local Government	(3) The Evaluation of Local Government Performance	(4) Farmers’ Trust in Local Government
Internet use	−2.813 *** (0.454)	−0.259 *** (0.079)	−0.037 *** (0.016)	−0.254 *** (0.074)
Views on people’s livelihood problems		0.011 *** (0.002)		
Evaluation of local government performance				0.978 *** (0.033)
Gender	−0.307 (0.320)	−0.264 *** (0.055)	−0.034 * (0.018)	−0.235 *** (0.052)
Age	0.126 *** (0.019)	0.019 *** (0.003)	0.005 *** (0.001)	0.016 *** (0.003)
Education	−1.675 *** (0.167)	0.000 (0.028)	0.046 *** (0.009)	−0.063 ** (0.026)
Health	0.476 *** (0.148)	0.167 *** (0.026)	0.060 *** (0.009)	0.113 *** (0.025)
Nationality	5.033 *** (1.421)	0.124 (0.234)	0.027 (0.071)	0.153 (0.213)
CPC member	−2.266 * (1.286)	−0.003 (0.244)	0.005 (0.082)	−0.033 (0.234)
NFC	−0.689 * (0.405)	−0.181 ** (0.071)	−0.025 (0.023)	−0.164 ** (0.066)
Religion	−0.368 (0.330)	0.097 * (0.057)	0.063 *** (0.019)	0.031 (0.054)
Trust in strangers	0.016 (0.079)	0.286 *** (0.013)	0.034 *** (0.004)	0.253 *** (0.012)
Per capita income	−0.015 (0.074)	−0.045 *** (0.011)	−0.002 (0.004)	−0.043 *** (0.011)
Per capita subsidy income	0.779 (0.560)	0.191 (0.142)	0.158 ** (0.080)	0.046 (0.092)
Family population	−0.173 ** (0.076)	0.011 (0.014)	0.009 ** (0.005)	0.001 (0.013)
Marriage (taking “unmarried” as reference)				
Married	−3.930 *** (0.446)	−0.716 *** (0.077)	−0.205 *** (0.026)	−0.560 *** (0.074)
Divorced	−4.691 *** (1.231)	−0.461 ** (0.212)	−0.136 * (0.070)	−0.380 * (0.194)
Widowed	−3.393 ** (1.395)	−0.133 (0.226)	0.025 (0.076)	−0.194 (0.216)
Region dummy	yes	yes	yes	yes
Constant	48.795 *** (1.308)	45.781 *** (1.796)	2.043 *** (0.075)	2.136 *** (0.222)
Observations	8754	8754	8754	8754
R2	0.080	0.089	0.034	0.182
Sobel test—Indirect effect	−0.031 *** (0.007)		−0.036 ** (0.016)	
Bootstrap test—Indirect effect	−0.031 *** (0.008)		−0.036 ** (0.016)	
Bootstrap 95% conf. interval	[−0.047, −0.016]		[−0.085, −0.013]	

Note: Robust standard errors are presented in parentheses; *, **, and *** indicate significance levels at 10%, 5%, and 1%, respectively.

**Table 7:** The chain mediating effect of views on people’s livelihood problems and government performance evaluation.

Variable Name	(1) The Evaluation of Local Government Performance	(2) Farmers’ Trust in Local Government
Internet use	−0.022 *** (0.006)	−0.237 *** (0.074)
Views on people’s livelihood problems	0.005 *** (0.001)	0.006 *** (0.002)
Evaluation of local government performance		0.968 *** (0.033)
Gender	−0.032 * (0.018)	−0.233 *** (0.052)
Age	0.004 *** (0.001)	0.015 *** (0.003)
Education	0.054 *** (0.009)	−0.052 ** (0.027)
Health	0.058 *** (0.009)	0.111 *** (0.025)
Nationality	0.001 (0.071)	0.122 (0.212)
CPC member	0.017 (0.081)	−0.019 (0.234)
NFC	−0.021 (0.023)	−0.160 ** (0.066)
Religion	0.065 *** (0.019)	0.034 (0.054)
Trust in strangers	0.034 *** (0.004)	0.253 *** (0.012)
Per capita income	−0.002 (0.004)	−0.043 *** (0.011)
Per capita subsidy income	0.154 * (0.079)	0.043 (0.092)
Family population	0.010 ** (0.005)	0.002 (0.013)
Marriage (taking “unmarried” as reference)		
Married	−0.185 *** (0.026)	−0.537 *** (0.074)
Divorced	−0.112 (0.069)	−0.352 * (0.194)
Widowed	0.042 (0.076)	−0.173 (0.217)
Region dummy	yes	yes
Constant	2.291 ***(0.082)	2.458 *** (0.243)
Observations	8754	8754
R2	0.041	0.183

Note: Robust standard errors are presented in parentheses; *, **, and *** indicate significance levels at 10%, 5%, and 1%, respectively.

## Data Availability

This paper used data from the China Family Panel Studies in 2018. CFPS collects data at the individual, family and community levels to reflect the changes in China’s society, economy, population, education and health, providing a data base for academic research and public policy analysis.
